# Reporting of financial conflicts of interest in meta-analyses of drug trials published in high-impact medical journals: comparison of results from 2017 to 2018 and 2009

**DOI:** 10.1186/s13643-020-01318-5

**Published:** 2020-04-08

**Authors:** Carla Benea, Kimberly A. Turner, Michelle Roseman, Lisa A. Bero, Joel Lexchin, Erick H. Turner, Brett D. Thombs

**Affiliations:** 1 Lady Davis Institute of the Jewish General Hospital, 4333 Cote Ste Catherine Road, Montreal, Quebec H3T 1E4 Canada; 2 Department of Psychiatry, McGill University, Montreal, Quebec Canada; 3 Kingsway Medical Centre Family Health Organization, Toronto, Ontario Canada; 4 Charles Perkins Centre and School of Pharmacy, Faculty of Medicine and Health, University of Sydney, Camperdown, New South Wales Australia; 5 School of Health Policy and Management, York University, Toronto, Ontario Canada; 6 Emergency Department, University Health Network, Toronto, Ontario Canada; 7 Behavioral Health and Neurosciences Division, VA Portland Health Care System, Portland, Oregon USA; 8 Department of Psychiatry, Oregon Health & Science University, Portland, Oregon USA; 9 Department of Epidemiology, Biostatistics, and Occupational Health, McGill University, Montreal, Quebec Canada; 10 Department of Medicine, McGill University, Montreal, Quebec Canada; 11 Biomedical Ethics Unit, McGill University, Montreal, Quebec Canada; 12 Department of Psychology, McGill University, Montreal, Quebec Canada; 13 Department of Educational and Counselling Psychology, McGill University, Montreal, Quebec Canada

**Keywords:** Financial conflicts of interest, Reporting, Meta-analysis, Research methods

## Abstract

**Background:**

A previous study found that 2 of 29 (6.9%) meta-analyses published in high-impact journals in 2009 reported included drug trials’ funding sources, and none reported trial authors’ financial conflicts of interest (FCOIs) or industry employment. It is not known if reporting has improved since 2009. Our objectives were to (1) investigate the extent to which pharmaceutical industry funding and author-industry FCOIs and employment from included drug trials are reported in meta-analyses published in high-impact journals and (2) compare current reporting with results from 2009.

**Methods:**

We searched PubMed (January 2017–October 2018) for systematic reviews with meta-analyses including ≥ 2 randomized controlled trials (RCTs) of patented drugs. We included 3 meta-analyses published January 2017–October 2018 from each of 4 high-impact general medicine journals, high-impact journals from 5 specialty areas, and the Cochrane Database of Systematic Reviews, as in the previous study.

**Results:**

Among 29 meta-analyses reviewed, 13 of 29 (44.8%) reported the funding source of included trials compared to 2 of 29 (6.9%) in 2009, a difference of 37.9% (95% confidence interval, 15.7 to 56.3%); this included 7 of 11 (63.6%) from general medicine journals, 3 of 15 (20.0%) from specialty medicine journals, and 3 of 3 (100%) Cochrane reviews. Only 2 of 29 meta-analyses (6.9%) reported trial author FCOIs, and none reported trial author-industry employment.

**Protocol Publication:**

A protocol was uploaded to the Open Science Framework prior to initiating the study. https://osf.io/8xt5p/

**Limitations:**

We examined only a relatively small number of meta-analyses from selected high-impact journals and compared results to a similarly small sample from an earlier time period.

**Conclusions:**

Reporting of drug trial sponsorship and author FCOIs in meta-analyses published in high-impact journals has increased since 2009 but is still suboptimal. Standards on reporting of trial funding described in the forthcoming revised PRISMA statement should be adapted and enforced by journals to improve reporting.

## Background

Financial conflicts of interest (FCOIs) in drug trials can influence trial design, drug dosages and comparators, data analysis, interpretation of findings, and the likelihood that favorable results are reported [1–7]. Industry-sponsored trials are approximately 30% more likely to report favorable efficacy results and conclusions than non-sponsored trials, and this is not explained by other trial elements associated with risk of bias [6]. Similarly, trials conducted by principal investigators with FCOIs have higher odds of reporting positive outcomes than trials led by non-affiliated principal investigators, controlling for trial funding source [7].

Meta-analyses are highly cited [8] and are prioritized in the development of clinical practice guidelines and in setting research priorities [9–11]. A review of a sample of 29 meta-analyses of drug trials published in high-impact medical journals in 2009, however, reported that only 2 (6.9%) reported funding sources and none reported author FCOIs or industry employment from included trials [12]. A 2012 review of 151 Cochrane reviews of drug trials found that only 46 (30.5%) reported the funding source of some or all included trials; only 16 (10.6%) provided any information on author FCOIs or industry employment [13].

In 2012, the Cochrane Collaboration began to require that funding sources and author FCOIs be reported for all trials included in Cochrane reviews [14–16]. The Preferred Reporting Items for Systematic Reviews and Meta-analyses (PRISMA) statement, which was published in 2009 [17, 18], however, did not address reporting of trial funding and author-industry financial ties from included trials. International Committee of Medical Journal Editors (ICMJE) guidelines require that meta-analysis authors declare their own FCOIs but do not address reporting of study funding or author FCOIs of included trials [19].

The objectives of this study were to (1) investigate the extent to which pharmaceutical industry funding and author-industry financial ties and employment from drug trials synthesized in meta-analyses are reported in meta-analyses published in high-impact journals and (2) compare current reporting with results from 2009 [12].

## Methods

Our study protocol was published on the Open Science Framework (https://osf.io/8xt5p/) and followed the methods of the previous Roseman et al. [12] study with 2 exceptions. First, whereas the previous study was limited to a 10-month period (January to October 2009), we extended the period to 22 months a priori (January 2017 to October 2018) to improve the likelihood that we could include the same number of meta-analyses from each journal or specialty area. Second, in addition to extracting reporting of trial information in meta-analyses, the previous study [12] reviewed FCOIs from all included trials. Since it is well-established that FCOIs are common in drug trials [1, 12, 20], we examined reporting in meta-analyses, but did not extract information from included trials.

### Study selection

To be included, publications had to include at least 1 meta-analysis that (1) was part of a documented systematic review, (2) statistically combined results from ≥ 2 RCTs, (3) did not include non-RCTs, (4) evaluated the efficacy or harm of a drug or class of drugs, and (5) included at least 1 drug in any study arm under patent in the USA at the time of publication based on the electronic US Food and Drug Administration Orange Book [21]. Drugs were defined broadly to include biologics and vaccines but not nutritional supplements (e.g., vitamins) or medical devices without a drug component. Status of potential drug products not found in the FDA database, such as products registered as drugs only outside of the USA, was determined by review of team members and consensus. A drug was considered under patent if any aspect of the active ingredient (e.g., dosage, route, strength) was protected by an unexpired patent. We included the patent requirement to restrict the sample to drugs of potential economic importance to pharmaceutical companies. See Additional Methods 1 for the title/abstract and full-text eligibility coding guides.

### Data sources and searches

We selected a sample of meta-analyses published from January 1, 2017, to October 25, 2018, in 3 categories of high-impact publications: (1) general medicine journals, (2) journals from 5 specialty medicine areas (oncology, cardiology, respiratory medicine, endocrinology, and gastroenterology), and (3) the Cochrane Database of Systematic Reviews [12].

In the previous study [12], among general medicine journals, we selected the 3 most recently published eligible meta-analyses from each journal with a 2008 impact factor ≥ 10 (*New England Journal of Medicine*, *JAMA*, *Lancet*, *BMJ*, *Annals of Internal Medicine*, *PLoS Medicine*) with fewer included if < 3 met eligibility criteria. Neither the New England Journal of Medicine nor PLoS Medicine had any eligible meta-analyses, and the Annals of Internal Medicine had only 2. Thus, in the present study, only meta-analyses from JAMA, Lancet, BMJ, and Annals of Internal Medicine were eligible, and we included only 2 meta-analyses from Annals of Internal Medicine.

In the previous study [12], we selected the top 5 specialty areas based on 2008 global pharmaceutical sales. For the present study, we included the same 5 specialty areas (oncology, cardiology, respiratory medicine, endocrinology, and gastroenterology). In each specialty area, we included the 3 most recently published eligible meta-analyses in the top impact factor journal in the specialty area based on the 2016 Clarivate Analytics Science Citation Index. If 3 eligible meta-analyses were not published in the top journal, we searched the journal with the next highest impact factor and continued in declining order of impact factor until 3 eligible meta-analyses were identified.

We searched MEDLINE via PubMed using limits of article type (“meta-analysis”) with journal names, supplemented by a manual search of each journal’s table of contents. Articles published online ahead of print, but not in the final format, as of the search date were not eligible. For articles published in the same journal issue, the one with the highest page number was considered most recent. In each Cochrane Database of Systematic Reviews issue, meta-analyses were reviewed in reverse sequence as the most recently published reviews are listed first.

### Data extraction

We uploaded search results into the systematic review software DistillerSR® for inclusion and exclusion and result coding. Review of identified articles from each general medicine journal, specialty medicine area, and the Cochrane Database of Systematic Reviews was conducted independently by 2 reviewers, 1 article at a time, in reverse temporal sequence until the targeted number of eligible meta-analyses was obtained. Any disagreements were resolved by consensus, involving a third reviewer if necessary.

Two investigators independently reviewed all included meta-analyses, including disclosure statements, article texts and tables, author bylines and acknowledgments, and online journal supplements to identify (1) in the meta-analysis: disclosed funding sources, author-industry financial ties, author-industry employment, and whether a quality or risk of bias assessment was conducted, and (2) whether or not the meta-analysis reported trial funding, author-industry financial ties, and author-industry employment from included drug RCTs. See Additional Methods 2 for the Meta-Analysis Data Extraction dictionary. Additionally, in February 2020, we examined author instructions from all journals with included meta-analyses to determine if they included instructions on reporting of funding sources, author-industry financial ties, or author-industry employment for studies included in meta-analyses published in the journal.

### Data synthesis and analysis

Study funding sources for meta-analyses were classified as pharmaceutical industry, non-industry (e.g., public granting agency, private not-for-profit granting agency), combined pharmaceutical industry and non-industry, no study funding, or not reported. Meta-analyses reported as funded “in part” by the pharmaceutical industry with no other indication of funding source or funded by a not-for-profit organization fully sponsored by pharmaceutical industry sources were coded as industry-funded. Industry funding was considered to be a provision of financial support, resources (e.g., statistical analyses), or study personnel.

Meta-analysis author financial ties to industry were defined per the ICMJE Uniform Disclosure Form for Potential Conflicts of Interest [19] and included current or former board membership, current or former consultancy, former industry employment, equity holdings (e.g., stock ownership, stock options), expert testimony, gifts, patents (planned, pending, issued), payment for manuscript preparation, other research funding, royalties, speaker fees/payment for presentation development, travel reimbursement, or other unspecified FCOIs, as disclosed in the article. If an article did not contain a disclosure statement, author-industry financial ties were coded as not reported. Authorship by persons employed by the pharmaceutical industry at the time of article publication was coded separately as “industry employment.”

For reporting in meta-analyses of trial funding, author-industry financial ties, and author-industry employment from included drug RCTs, for each category, we determined if the meta-analysis reported for all, some, or no included RCTs. When information was reported, we determined where information could be found in the meta-analysis (e.g., text, characteristics of studies table, risk of bias assessment, footnote).

Any discrepancies in data extraction were resolved by consensus, including consultation with a third reviewer if necessary.

We reported descriptive characteristics of included meta-analyses, their funding, and author-industry financial ties. To compare the proportion of meta-analyses that reported study funding, author financial ties, and author employment from included RCTs, we generated 95% confidence intervals (CIs) for the differences in proportions [22].

### Role of the funding source

No funder had any role in study design; in the collection, analysis, and interpretation of data; in the writing of the report; or in the decision to submit the paper for publication. Dr. Thombs had full access to all data in the study and had final responsibility for the decision to submit for publication.

## Results

### Article selection

**Fig. 1:**
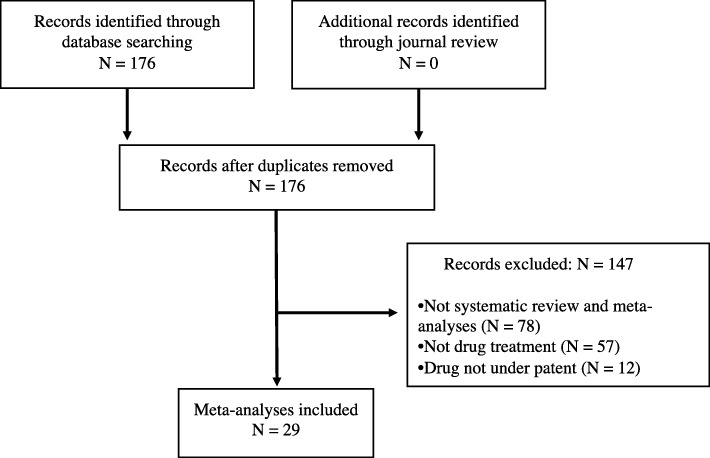
PRISMA flowchart of included meta-analyses

A total of 176 publications were reviewed (37 from general medicine journals, 121 from specialty medicine journals, 18 from the Cochrane Database of Systematic Reviews) to obtain the 29 that were included in the review (Fig. 1) [23–51]. As shown in Table 1, impact factors of journals with included publications ranged from 17.2 to 47.8 in general medicine, 11.9 to 24.0 in oncology, 19.3 to 19.9 in cardiology, 10.3 to 10.6 in respiratory medicine, 11.9 to 19.7 in endocrinology, 16.7 to 18.4 in gastroenterology, and 6.3 for the Cochrane Database of Systematic Reviews. The 29 selected meta-analyses evaluated a broad spectrum of pharmacological interventions, including 11 on treatment efficacy [24, 29, 33, 35, 36, 40, 42, 44–47], 2 on harms [31, 50], and 16 [23, 25, 28, 30, 32, 34, 37–39, 41, 43, 48, 49, 51] on both efficacy and harms. Between 2 and 522 RCTs were included in each meta-analysis. None of the journals with included meta-analyses mentioned reporting of funding or author FCOI of studies included in meta-analyses that are published in the journal.

**Table 1:** Characteristics of included meta-analyses

Author	Journal/year	2016 impact factor	Number of drug RCTs in meta-analysis	Publication dates of articles reviewed	Setting/diagnosis	Pharmacological intervention	Comparison arm(s)	Efficacy/harm	Funding source	Number of authors with disclosed industry financial ties/number of authors	Number of authors employed by industry/number of authors
**General medicine**											
Feller et al. [23]	JAMA/2018	44.4	21	1984–2017	Non-pregnant adults with subclinical hypothyroidism	Thyroid hormone therapy	Placebo, no intervention	Efficacy and harm	Non-industry	0/15	0/15
McIntyre et al. [24]	JAMA/2018	44.4	23	1999–2017	Patients with distributive shock	Vasopressin—4 included plus catecholamine vasopressors	Catecholamines vasopressors—2 included	Efficacy	Not reported	2/10	0/10
Zheng et al. [25]	JAMA/2018	44.4	236	2004–2017	Patients with type 2 diabetes	NMA: SGLT-2 inhibitors, GLP-1 agonists, DPP-4 inhibitors	Placebo, no treatment	Efficacy and harm	Not reported	1/7	0/7
Cipriani et al. [26]	Lancet/2018	47.8	522	1979–unpublished	Adults with major depressive disorder	NMA: antidepressants—21 included	Placebo	Efficacy and harm	Non-industry	4/18	0/18
Gayet-Ageron et al. [27]	Lancet/2018	47.8	2	2010–2017	Patients with acute severe hemorrhage	Tranexamic acid	Placebo	Efficacy and harm	Not reported	0/6	0/6
Jinatongthai et al. [28]	Lancet/2017	47.8	40	1986–2005	Patients with ST-segment elevation myocardial infarction	NMA: fibrinolytic agents—4 included, parenteral anticoagulants—4 included, Gp IIb/IIIa inhibitors—3 included, antiplatelets—3 included	N/A	Efficacy and harm	No study funding	0/8	0/8
Alibhai et al. [29]	Ann Intern Med/2017	17.2	23^a^	2001–2014	Men with non-metastatic prostate cancer	Bisphosphonates—5 included	Placebo	Efficacy	Non-industry	3/9	0/9
Wilson et al. [30]	Ann Intern Med/2017	17.2	10^b^	2003–2012	Adults with chronic kidney disease	Bisphosphonates—4 included, raloxifene, denosumab, teriparatide	Placebo, no treatment, active control	Efficacy and harm	Non-industry	0/8	0/8
Baxi et al. [31]	BMJ/2018	20.8	13	2015–2017	Patients with cancer with recurrent or metastatic disease	AntiPD-1—3 included	Chemotherapy drug, small molecule inhibitor, investigators’ choice	Harm	Non-industry	0/7	0/7
López-López et al. [32]	BMJ/2017	20.8	23	1989–2014	Adults with non-valvular atrial fibrillation eligible for oral anticoagulation	DOACs—5 included	Warfarin or other vitamin K antagonist, aspirin, clopidogrel	Efficacy and harm	Non-industry	0/18	0/18
Sadeghirad et al. [33]	BMJ/2017	20.8	10	1993–2017^c^	Patients with sore throat	Corticosteroids—3 included	Placebo, standard care	Efficacy	No study funding	0/9	0/9
**Specialty journals**											
**Oncology**											
McCarthy et al. [34]	J Clin Oncol/2017	24.0	3	2012–2014	Patients with newly diagnosed multiple myeloma	Lenalidomide	Placebo or observation	Efficacy and harm	Industry-financial support and resource^d^	15/19	4/19
van Beurden-Tan et al. [35]	J Clin Oncol/2017	24.0	17	2005–2016	Adults with relapsed and/or refractory multiple myeloma	NMA: 18 treatments for multiple myeloma	N/A	Efficacy	Non-industry	5/5	0/5
Abdel-Qadir et al. [36]	Ann Oncol/2017	11.9	16	1983–2013	Adults with cancer	NMA: dexrazoxane, angiotensin antagonist, beta-blockers, statins, co-enzyme Q-10, prenylamine, N-acetylcysteine	Placebo or no treatment	Efficacy	Not reported	0/7	0/7
**Cardiology**											
Siontis et al. [37]	Circulation/2017	19.3	4	2014–2016	Patients with valvular heart disease	DOAC—4 included	Warfarin	Efficacy and harm	Not reported	1/4	0/4
Renda et al. [38]	J Am Coll Cardiol/2017	19.9	4	2009–2013	Patients with atrial fibrillation and valvular heart disease	NOAC—4 included	Warfarin	Efficacy and harm	Not reported	3/4	0/4
Lau et al. [39]	J Am Coll Cardiol/2017	19.9	7	2007–2015	Patients with coronary artery disease	Potent P2Y12 inhibitors—3 types included	Placebo or clopidogrel	Efficacy and harm	Not reported	13/16	0/16
**Respiratory medicine**											
Verberkt et al. [40]	Eur Respir J/2017	10.6	22^e^	1982–2015	Patients with chronic breathlessness due to advanced disease	Opioids—8 included	Placebo	Efficacy	Non-industry	3/9	0/9
Ding et al. [41]	J Thorac Oncol/2017	10.3	16	2008–2015	Treatment-naive patients with advanced EGFR-mutant non-small cell lung cancer	EGFR TKI—3 included	EGFR TKI—3 included	Efficacy and harm	Not reported	0/8	0/8
Lee et al. [42]	J Thorac Oncol/2017	10.3	3	2015–2016	Patients with metastatic EGFR-mutated non-small cell lung cancer	Immune checkpoint inhibitors—3 included	Docetaxel	Efficacy	Not reported	0/7	0/7
**Endocrinology**											
Bethel et al. [43]	Lancet Diabetes Endocrinol/2018	19.7	4	2015–2017	Adults with type 2 diabetes	GLP-1 receptor agonists—4 included	Placebo	Efficacy and harm	Industry-financial support	15/17	3/17
de Carvalho et al. [44]	Diabetes Care/2018	11.9	20	2012–2016	Participants with familial or non-familial hypercholesterolemia	PCSK9 inhibitors—3 included	Placebo, ezetimibe, atorvastatin, rosuvastatin, standard therapy	Efficacy	Not reported	0/3	0/3
Maiorino et al. [45]	Diabetes Care/2017	11.9	26	2011–2016	Adults with type 2 diabetes	Insulin regimens plus GLP-1 receptor agonists	Insulin regimens	Efficacy	Not reported	2/6	0/6
**Gastroenterology**											
Khera et al. [46]	Gastroenterology/2018	18.4	28	1998–2015	Obese or overweight adults	NMA: orlistat, lorcaserin, naltrexone-bupropion, liraglutide, phentermine-topiramate	Alternate treatment or placebo	Efficacy	No study funding	0/9	0/9
Nelson et al. [47]	GUT/2017	16.7	21	2003–2015	Adults with chronic idiopathic constipation	NMA: diphenyl methanes or derivatives—2 included, 5-HT4 receptor agonists—3 included, GC-C receptor agonist—1 included, chloride channel type 2 opener—1 included, apical sodium bile acid—1 included	N/A	Efficacy	Not reported	1/9	0/9
Ford et al. [48]	GUT/2017	16.7	13	1986–2015	Adults (participants aged > 16 years) with functional dyspepsia	Psychotropic drugs—6 classes	Placebo	Efficacy and harm	Not reported	0/6	0/6
**Cochrane reviews**											
Tenforde et al. [49]	Cochrane Database Syst Rev/2018	6.3	13	1997–2018	Adults with HIV-associated cryptococcal meningitis	NMA: antifungal drugs, adjunctive drugs	N/A	Efficacy and harm	Non-industry	1/7	0/7
McNicol et al. [50]	Cochrane Database Syst Rev/2018	6.3	11^f^	1992–2012	Pediatric patients with postoperative pain	Ketorolac	Placebo, opioid	Harm	Non-industry	1/3	0/3
Normansell et al. [51]	Cochrane Database Syst Rev/2018	6.3	4^g^	1974-2016	Adults and children with exacerbations of asthma	Macrolide antibiotics—3 included, penicillin—2 included	Placebo	Efficacy and harm	Non-industry	0/6	0/6

*5-HT4* 5-hydroxytryptamine receptor 4, *DOAC* direct-acting oral anticoagulant, *DPP-4* dipeptidyl peptidase 4, *EGFR* epidermal growth factor receptor, *GC-C* guanylate cyclase C, *GLP-1* glucagon-like peptide-1, *Gp IIb/IIIa* glycoprotein IIb/IIIa, *N/A* not applicable (no placebo or no treatment arm in NMA), *NMA* network meta-analysis, *NOAC* non-vitamin K antagonist oral anticoagulants, *PCSK9* proprotein convertase subtilisin/kexin type 9, *PD-1* programmed cell death protein 1, *SGLT-2* sodium-glucose cotransporter 2, *TKI* tyrosine kinase inhibitors

^a^The systematic review included 27 RCTs in total, of which 23 had their results pooled

^b^The systematic review included 13 RCTs in total, of which 10 had their results pooled

^c^The date for 1 of the included RCTs was reported as 1994 in a single figure and 1993 in all other instances; therefore, 1993 was used as the beginning of the date range of included studies

^d^The systematic review included an acknowledgment thanking Kristina Hernandez and Peter Simon for medical writing assistance, sponsored by Celgene Corporation, which was coded as industry funding in the form of resources

^e^The systematic review included 35 RCTs in total, of which 22 had their results pooled

^f^The systematic review included 13 RCTs in total, of which 11 had their results pooled

^g^The systematic review included 6 RCTs in total, of which 4 had their results pooled

### Study funding and author-industry financial ties of meta-analyses

As shown in Tables 1 and 2 of 29 (6.9%) included meta-analyses, both published in specialty journals [34, 43], reported receiving pharmaceutical industry funding, 11 (37.9%) reported non-industry funding [23, 26, 29–32, 35, 40, 49–51], 3 reported no study funding (10.3%) [28, 33, 46], and the funding source of 13 (44.8%) was not reported [24, 25, 27, 36–39, 41, 42, 44, 45, 47, 48]. Meta-analysis funding sources were reported for 8 of 11 meta-analyses from general medicine journals (72.7%) [23, 26, 28–33], 5 of 15 (33.3%) from specialty medicine journals [34, 35, 40, 43, 46], and all 3 (100%) Cochrane reviews [49–51].

**Table 2:** Financial ties to the pharmaceutical industry among authors of reviewed meta-analyses

Author	Journal	Number of authors	Authors that reported any industry financial ties	Authors that reported industry board membership	Authors that reported industry consultancy	Authors that reported equity holdings	Authors that reported receiving research funding from industry	Authors that reported holding patents	Authors that reported receiving royalties	Authors that reported receiving speaker fees/payment for development of presentations	Authors that reported receiving travel reimbursement	Authors that reported receiving unspecified FCOI
**General medicine**												
Feller et al. [23]	JAMA	15	0/15	0	0	0	0	0	0	0	0	0
McIntyre et al. [24]	JAMA	10	2/10	0	0	0	2	0	0	0	0	1
Zheng et al. [25]	JAMA	7	1/7	1	0	0	0	0	0	1	1	0
Cipriani et al. [26]	Lancet	18	4/18	0	2	0	1	0	0	4	0	0
Gayet-Ageron et al. [27]	Lancet	6	0/6	0	0	0	0	0	0	0	0	0
Jinatongthai et al. [28]	Lancet	8	0/8	0	0	0	0	0	0	0	0	0
Alibhai et al. [29]	Ann Intern Med	9	3/9	1	1	0	1	0	0	0	0	1^a^
Wilson et al. [30]	Ann Intern Med	8	0/8	0	0	0	0	0	0	0	0	0
Baxi et al. [31]	BMJ	7	0/7	0	0	0	0	0	0	0	0	0
López-López et al. [32]	BMJ	18	0/18	0	0	0	0	0	0	0	0	0
Sadeghirad et al. [33]	BMJ	9	0/9	0	0	0	0	0	0	0	0	0
**Total—general medicine**			4/11									
**Specialty journals**												
**Oncology**												
McCarthy et al. [34]	J Clin Oncol	19	15/19	0	8	3	4	1	1	0	2	8
van Beurden-Tan et al. [35]	J Clin Oncol	5	5/5	0	3	0	5	0	0	0	0	1
Abdel-Qadir et al. [36]	Ann Oncol	7	0/7	0	0	0	0	0	0	0	0	0
**Cardiology**												
Siontis et al. [37]	Circulation	4	1/4	1	0	0	0	0	0	0	0	0
Renda et al. [38]	J Am Coll Cardiol	4	3/4	0	2	0	2	0	0	1	0	2
Lau et al. [39]	J Am Coll Cardiol	16	13/16	2	8	0	12	1	0	4	1	2^b^
**Respiratory medicine**												
Verberkt et al. [40]	Eur Respir J	9	3/9^c^	1	2	0	2	0	0	2	0	0
Ding et al. [41]	J Thorac Oncol	8	0/8	0	0	0	0	0	0	0	0	0
Lee et al. [42]	J Thorac Oncol	7	0/7	0	0	0	0	0	0	0	0	0
**Endocrinology**												
Bethel et al. [43]	Lancet Diabetes Endocrinol	17	15/17	5^d^	2	2	8	0	0	2	1	7^e^
de Carvalho et al. [44]	Diabetes Care	3	0/3	0	0	0	0	0	0	0	0	0
Maiorino et al. [45]	Diabetes Care	6	2/6	0	2	0	0	0	0	2	0	0
**Gastroenterology**												
Khera et al. [46]	Gastroenterology	9	0/9	0	0	0	0	0	0	0	0	0
Nelson et al. [47]	GUT	9	1/9	0	1	0	1	0	0	0	0	0
Ford et al. [48]	GUT	6	0/6	0	0	0	0	0	0	0	0	0
**Total—specialty:**			9/15									
**Cochrane reviews**												
Tenforde et al. [49]	Cochrane Database Syst Rev	7	1/7	0	0	0	1	0	0	0	0	0
McNicol et al. [50]	Cochrane Database Syst Rev	3	1/3	0	0	0	0	0	0	0	1	0
Normansell et al. [51]	Cochrane Database Syst Rev	6	0/6	0	0	0	0	0	0	0	0	0
**Total Cochrane**			2/3									

^a^One author reported receiving “personal fees” from industry but did not specify further, and this was coded as unspecified FCOI

^b^One author reported “consulting fees, honoraria, or both” which was coded as unspecified FCOI

^c^Only 5 authors out of 9 provided ICMJE forms for disclosure of potential FCOIs in the supplemental material; the information was not reported elsewhere

^d^Being a member of the board for the non-profit organization “AstraZeneca HealthCare Foundation” was considered as industry board membership

^e^Four authors reported receiving “personal fees” but did not specify further, 1 author reported receiving “grants or honoraria for consultancy or lectures,” 1 reported “honorarium for steering committee attendance,” 1 reported honoraria for “participation in study committees,” and 1 reported honoraria for “participation in academic conferences”; these were all coded as unspecified FCOIs

All 29 meta-analyses included author COI statements. In 15 of the 29 meta-analyses (51.7%), at least 1 author reported 1 or more financial ties to the pharmaceutical industry [24–26, 29, 34, 35, 37–40, 43, 45, 47, 49, 50], whereas all authors reported no financial ties in 14 (48.3%) [23, 27, 28, 30–33, 36, 41, 42, 44, 46, 48, 51]. In 5 of 29 (17.2%) meta-analyses [34, 35, 38, 39, 43], all published in specialty medicine journals and including the 2 meta-analyses with industry funding [34, 43], the majority of authors had financial ties to industry. Author-industry financial ties were present in 4 of 11 meta-analyses published in general medicine journals (36.4%) [24–26, 29], 9 of 15 (60.0%) in specialty medicine journals [34, 35, 37–40, 43, 45, 47], and 2 of 3 (66.7%) Cochrane reviews [49, 50]. Specific types of author ties to industry are shown in Table 2.

### Reporting of trial funders and author FCOI from RCTs included in meta-analyses

As shown in Table 3, 13 of 29 (44.8%) meta-analyses reported the funding sources of included RCTs; 12 reported for all included RCTs [23, 24, 26, 27, 29, 30, 32, 34, 45, 49–51], whereas 1 reported for 5 of 7 included RCTs [39]. Funding sources of included RCTs were reported for 7 of 11 (63.6%) meta-analyses from general medicine journals [23, 24, 26, 27, 29, 30, 32], 3 of 15 (20.0%) from specialty medicine journals [34, 39, 45], and for all 3 (100%) Cochrane reviews [49–51]. The mean, median, and range of the number of RCTs in meta-analyses that reported trial funding sources were 52.9, 13, and from 2 to 522, respectively. For those that did not report funding sources, they were 29.2, 16, and from 3 to 236, respectively.

**Table 3:** Disclosure and reporting in meta-analyses of randomized controlled trial funding source, author financial ties to the pharmaceutical industry, and author employment by the pharmaceutical industry

Author	Journal	Number of meta-analyzed RCTs	Meta-analysis reported RCT funding	Place in publication of RCT funding	Meta-analysis reported RCT author-industry financial ties	Place in publication of RCT author-industry financial ties	Meta-analysis reported RCT author-industry employment	Quality or risk assessment method of meta-analysis
**General medicine**								
Feller et al. [23]	JAMA	21	Yes, for each included study	Text (as summary statement—for some but not all included studies—RCTs referenced) Characteristics of included studies table (in article—for each included study)	No	N/A	No	Cochrane
McIntyre et al. [24]	JAMA	23	Yes, for each included study	Characteristics of included studies table (in supplementary material—web link only—no mention in text)	Yes, for some but not all included studies^a^	Characteristics of included studies table (in supplementary material—web link only—no mention in text)	No	Cochrane
Zheng et al. [25]	JAMA	236	No	N/A	No	N/A	No	Cochrane
Cipriani et al. [26]	Lancet	522	Yes, for each included study^b^	Text (as summary statement—RCTs not referenced) Characteristics of included studies (in supplementary material—web link only—funder name not mentioned)	No^c^	N/A	No	Cochrane
Gayet-Ageron et al. [27]	Lancet	2	Yes, for each included study	Abstract	No	N/A	No	Cochrane
Jinatongthai et al. [28]	Lancet	40	No	N/A	No	N/A	No	Cochrane
Alibhai et al. [29]	Ann Intern Med	23	Yes, for each included study	Risk of bias assessment text section (as summary statement—for some but not all included studies—RCTs not referenced) Risk of bias assessment table (in article—for each included study)	No	N/A	No	Cochrane
Wilson et al. [30]	Ann Intern Med	10	Yes, for each included study^d^	Risk of bias assessment text section (as summary statement—RCTs referenced) Characteristics of studies included table (in article—for each included study)	No	N/A	No	Cochrane
Baxi et al. [31]	BMJ	13	No	N/A	No	N/A	No	Cochrane
López-López et al. [32]	BMJ	23	Yes, for each included study	Text (as summary statement—RCTs not referenced) Characteristics of included studies table (for each included study—in supplementary material—web link only)	No	N/A	No	Cochrane
Sadeghirad et al. [33]	BMJ	10	No	N/A	No	N/A	No	Cochrane
**General medicine total**		**923**	**7/11 (63.6%)**		**1/11 (9.1%)**		**0/11 (0.0%)**	
**Specialty medicine**								
**Oncology**								
McCarthy et al. [34]	J Clin Oncol	3	Yes, for each included study	“Support” section at end of article	No	N/A	No	None
van Beurden-Tan et al. [35]	J Clin Oncol	17	No	N/A	No	N/A	No	None
Abdel-Qadir et al. [36]	Ann Oncol	16	No	N/A	No	N/A	No	Cochrane
**Cardiology**								
Siontis et al. [37]	Circulation	4	No	N/A	No	N/A	No	None
Renda et al. [38]	J Am Coll Cardiol	4	No	N/A	No	N/A	No	Cochrane
Lau et al. [39]	J Am Coll Cardiol	7	Yes, for some but not all studies^e^	Footnote on first 3 pages of article	No	N/A	No	None
**Respiratory medicine**								
Verberkt et al. [40]	Eur Resp J	22	No	N/A	No	N/A	No	Cochrane
Ding et al. [41]	J Thorac Oncol	16	No	N/A	No	N/A	No	None
Lee et al. [42]	J Thorac Oncol	3	No	N/A	No	N/A	No	None
**Endocrinology**								
Bethel et al. [43]	Lancet Diabetes Endocrinol	4	No	N/A	No	N/A	No	Jadad
de Carvalho et al. [44]	Diabetes Care	20	No	N/A	No	N/A	No	Cochrane
Maiorino et al. [45]	Diabetes Care	26	Yes, for each included study	Text (as summary statement—RCTs not referenced) Characteristics of included studies table (in article—for each included study)	No	N/A	No	Cochrane, Jadad
**Gastroenterology**								
Khera et al. [46]	Gastroenterology	28	No	N/A	No	N/A	No	Cochrane
Nelson et al. [47]	Gut	21	No	N/A	No	N/A	No	Cochrane
Ford et al. [48]	Gut	13	No	N/A	No	N/A	No	Cochrane
**Specialty medicine total:**		**204**	**3/15 (20.0%)**		**0/15 (0.0%)**		**0/15 (0.0%)**	
**Cochrane database of systematic reviews**								
Tenforde et al. [49]	Cochrane Database Syst Rev	13	Yes, for each included study	Risk of bias assessment in main text (as a summary statement—for some but not all included studies—RCTs referenced)^f^ Risk of bias assessment table in characteristics of included studies section (supplementary material—in article—for some but not all included studies)^g^ Characteristics of included studies table (supplementary material—in article—for each included study)	Yes, for each included study	Risk of bias assessment in main text (as a summary statement—for some but not all included studies—studies referenced)^f^ Risk of bias assessment table in characteristics of included studies section (supplementary material—in article—for some but not all included studies)^h^ Characteristics of included studies table (supplementary material—in article—for each included study)	No	Cochrane
McNicol et al. [50]	Cochrane Database Syst Rev	11	Yes, for each included study	Characteristics of included studies table (supplementary material—in article)	No	N/A	No	Cochrane
Normansell et al. [51]	Cochrane Database Syst Rev	4	Yes, for each included study	Text (as a summary statement—RCTs referenced) Characteristics of included studies table (supplementary material—in article—for each included study)	No^i^	N/A	No	Cochrane
**Cochrane total**		**28**	**3/3 (100%)**		**1/3 (33.3%)**		**0/3 (0.0%)**	
**Total**		**1155**	**13/29 (44.8%)**		**2/29 (6.9%)**		**0/29 (0.0%)**	

*FCOI* financial conflict of interest, *N/A* not applicable, *RCT* randomized controlled trial

^a^Author FCOIs are reported for 21 out of 23 RCTs. Reporting of “All authors submitted the ICMJE Form for Disclosure” for 1 study was not considered reporting of author FCOIs. Reporting of “Funding source: Ferring pharmaceuticals, patents related to the use of vasopressin in septic shock” for 1 study was not considered reporting of author FCOIs since not specified and was only coded as RCT funding source reported

^b^The authors considered funding for included studies as “sponsored” when it was indicated anywhere in the text that the study was funded/sponsored by the company which manufactured or marketed the drug in question, or if 1 or more of the authors were affiliated with the company in question, or if the data came from the documents provided by or obtained from the company website. Sponsorship was rated as “unclear” if the authors only listed the names of the companies in question in their declaration of conflicts of interest. Names of the pharmaceutical companies that sponsored trials were not reported

^c^Author FCOIs with manufacturer of researched drug, among funding from manufacturer, or data obtained from manufacturer, qualified a study of “sponsored,” but no further specification was given. A study being “sponsored” was only coded as having funding sources reported

^d^Authors reported RCT funding of industry, not industry, or not reported, and sponsors’ names were not mentioned

^e^Funding was reported for 5 out of 7 included RCTs

^f^In risk of bias assessment text section, the summarized “other bias” optional Cochrane domain included both direct support from pharmaceutical manufacturers of study drugs and authors receiving research support from manufacturers without the role of the drug companies clearly stated. Studies are referenced, but it is not mentioned for which FCOI (direct support from industry and/or author support from industry)

^g^In the “other bias” optional Cochrane domain in risk of bias assessment table, located in the characteristics of included studies section, funding is only reported for RCTs sponsored by pharmaceutical industry, with no mention of the name of the pharmaceutical company, which is only mentioned in the “Notes” section of the characteristics of included studies table

^h^In “other bias” optional Cochrane domain in risk of bias assessment table, located in the characteristics of included studies section, author FCOIs are only reported for RCTs where FCOIs are listed

^i^Authors stated that they extracted data on “notable conflicts of interest of trial authors” but no information on included RCTs author FCOIs was reported in the text or supplementary materials

**Fig. 2:**
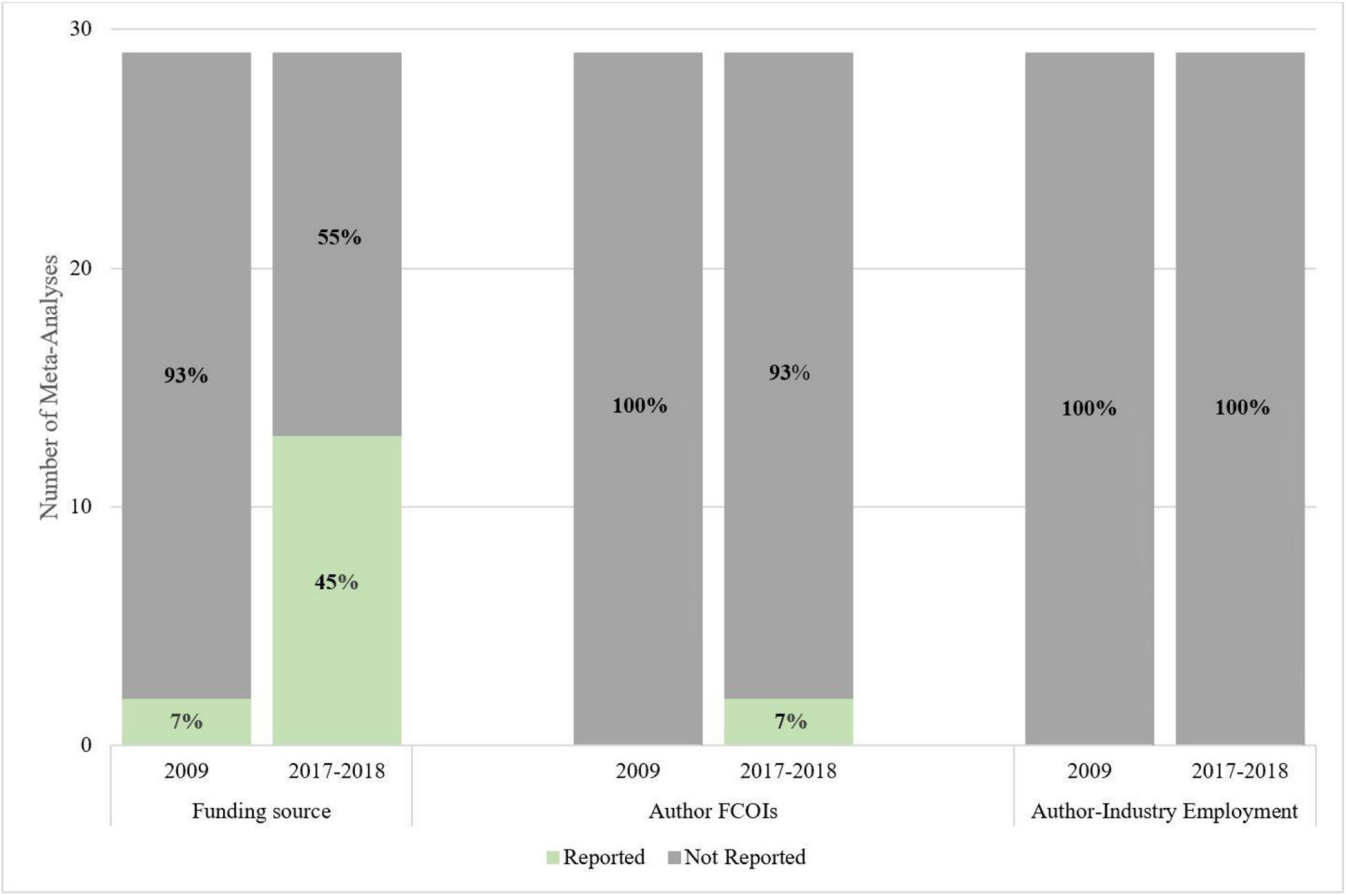
Percentage of included meta-analyses that reported trial funding, author financial conflict of interest, and author-industry employment for 2009 and 2017–2018

Only 2 of the 29 meta-analyses (6.9%) reported trial author-industry financial ties, including 1 from a general medicine journal [24] and 1 Cochrane review [49]. None of the 29 meta-analyses reported industry employment status of included RCT authors. See Fig. 2.

### Comparison of reporting of industry funding of included RCTs in 2017–2018 versus 2009

The overall percentage of meta-analyses of drug treatments that reported the funding source of included RCTs increased from 6.9% (2 of 29) in 2009 to 44.8% (13 of 29) in 2017–2018, a difference of 37.9% (95% CI, 15.7 to 56.3%). This included an increase from 0% (0 of 11) to 63.6% (7 of 11) in general medicine journals (difference 63.6%; 95% CI, 25.3 to 84.8%), an increase from 6.7% (1 of 15) to 20.0% (3 of 15) in specialty medicine journals (difference 13.3%; 95% CI, − 13.2 to 39.1%), and an increase from 1 of 3 (33.3%) to 3 of 3 (100%) among Cochrane reviews.

## Discussion

The main finding was that the reporting of funding sources of drug trials included in meta-analyses in high-impact journals improved, from 2 of 29 (6.9%) in 2009 to 13 of 29 (44.8%) in 2017–2018, but it continues to be sub-optimal. Only 2 of 29 (6.9%) meta-analyses provided information on author-industry financial ties from included trials, and no meta-analyses reported if industry employees were involved in the trials.

In 2012, the Cochrane Collaboration began to require that trial funding sources and conflicts of interest of authors of included trials be reported in the “characteristics of included studies” table of all Cochrane reviews [15], and this is still mandatory [16]. We evaluated 3 Cochrane reviews, and all 3 reported trial funding sources, but only 1 of 3 provided information on author-industry financial ties from included trials. A recent study [52] that investigated the extent to which recently published meta-analyses reported trial funding, author-industry financial ties, and author-industry employment from included RCTs found that reporting of trial funding in Cochrane meta-analyses increased from 30% (46 of 151 reviews) in 2010 to 84% (90 of 107) in 2016–2018. Reporting of trial author-industry financial ties increased from 7% (11 of 151) in 2010 to 44% (47 of 107) in 2016–2018, which suggests that this could still improve. Non-Cochrane meta-analyses published in 2016–2018 reported funding sources of included studies 15% of the time (21 of 143) and author-industry financial ties from included trials 1% of the time (2 of 143).

Cochrane reviews are recognized for their rigor [53] and often seen as the standard for systematic reviews on the benefits and harms of health care interventions [54, 55]. Consistent with this, Cochrane reporting standards are highlighted on the PRISMA website [56]. Ideally, the Cochrane Collaboration would ensure that authors of reviews adhere to both the requirement to report funding of trials included in reviews and to report author-industry financial ties from those trials. Nonetheless, Cochrane provides an example of how institutional commitment can lead to change on a large scale, which suggests that other journals could achieve similar results, but that it would require explicit guidance from journal editors and enforcement of that guidance.

The original PRISMA statement, which was published in 2009, did not address reporting of the funding sources of studies included in systematic reviews and meta-analyses or the FCOIs of study authors [17, 18]. An updated PRISMA statement is forthcoming and, though not completed, based on a preliminary version will likely encourage, though not required, reporting of funding and author FCOI from studies included in systematic reviews and meta-analyses (personal communication, David Moher, February 13, 2020). It is possible that including encouragement to report on funding and FCOI in PRISMA could improve reporting, but the lack of a strong requirement and inclusion as an item in the checklist itself and the general low adherence to PRISMA [57] suggests that this may not have a strong effect.

It is not clear why general medicine journals improved in reporting between 2009 and the present study. It is unlikely related to PRISMA, since the existing PRISMA statement does not touch upon this issue. It is possible that this may be due to a more general awareness of these issues, that Cochrane added this requirement for its reviews, or a higher scrutiny by editors of these journals than previously or compared to specialty journals. None of the general medicine or specialty journals included in our review mention the reporting of funding sources or author FCOI from studies included in systematic reviews in their instructions to authors. Ideally, the forthcoming PRISMA checklist would include funding and FCOI of studies included in systematic reviews and meta-analyses as a dedicated item, which could support improvements in reporting, if adopted and enforced by reviewers and editors.

We previously recommended that the Cochrane Risk of Bias tool [58] be revised to include risk of bias due to industry sponsorship of trials and FCOIs of trial investigators [12]. This would be consistent with empirical evidence that has linked both sponsorship and other FCOIs to trial outcomes, controlling for other factors known to be associated with bias [6, 7, 14]. There is no, however, consensus on this approach [14, 59]. Currently, an alternative is being created to explicitly address risk of bias from industry sponsorship of trials and author-industry financial ties in Cochrane reviews, the Tool for Addressing Conflicts of Interest in Trials (TACIT) [60]. Once completed, TACIT will include a Conflicts of Interest Grid, which will facilitate a systematic collection of relevant information and allow for determination of when there is notable concern, which may then be integrated into an assessment of risk of bias. In the present study, only 3 meta-analyses, 1 of which was a Cochrane review, attempted to incorporate funding sources of included trials into an assessment of risk of bias.

In interpreting results from this study, there are limitations to consider. First, the focus of the study was on reporting of trial funding and trial author FCOIs, and it was not designed to assess whether these were associated with meta-analysis quality or with the results of meta-analyses. Second, in replicating the methods of the previous study from 2009 [12], we selected 29 meta-analyses from high-impact journals in general medicine and 5 specialty areas for review; thus, it is not known to what degree these results may be generalizable to other areas of medicine or to lower impact journals. Third, we examined only a relatively small number of meta-analyses and compared results to a similarly small sample from an earlier time period.

## Conclusion

In summary, reporting of funding sources of included trials in meta-analyses of drug treatments published in high-impact journals has improved since 2009 but is still alarmingly low. Fewer than half of the meta-analyses we reviewed reported funding sources of included trials, and fewer than a third provided information on trial funding in the main meta-analysis report. Reporting of trial author FCOIs and industry employment is even more concerning. Only 2 studies reported trial author-industry financial ties, and none directly reported whether industry employees were authors of included trials. Confidence in medical research and the quality of care delivered by those who rely on evidence from meta-analyses depends on transparent reporting and the ability to evaluate the degree to which conflicts of interest may have influenced trial design, conduct, and outcomes. The forthcoming revised PRISMA statement will require transparent reporting of funding in trials included in systematic reviews and meta-analyses, and the new TACIT tool is being developed by the Cochrane Collaboration to supplement its risk of bias tool and to integrate considerations of FCOIs into bias assessment. We encourage uptake of both of these tools by journals and authors of systematic reviews and meta-analyses so that the potential influence of industry sponsorship and other author-industry ties can be considered by users of systematic reviews and meta-analyses.

## Supplementary information

**Additional file 1:.** Methods 1. Title/Abstract and Full Text Eligibility Coding Guide.

**Additional file 2:.** Methods 2. Meta-Analysis Data Extraction.

## Data Availability

All data extracted during this study are provided in Tables 1, 2 and 3.

## Abbreviations

FCOI: Financial conflicts of interest
RCT: Randomized controlled trial
