# Supplementary material for: Reporting of financial conflicts of interest in meta-analyses of drug trials published in high-impact medical journals: comparison of results from 2017 to 2018 and 2009
Source: Syst Rev. 2020 Apr 8;9:77. doi: 10.1186/s13643-020-01318-5 (PMC7140556; doi:10.1186/s13643-020-01318-5)
Supplement: Supplementary file 1 — Additional file 1:. Methods 1. Title/Abstract and Full Text Eligibility Coding Guide. [file 13643_2020_1318_MOESM1_ESM.docx]

**Additional Methods 1. Title/Abstract and Full Text Eligibility Coding Guide**

Title/Abstract:

**Is this potentially an eligible meta-analysis that states in the abstract that it is reporting pooled results of RCTs of a patented drug?**

**No: Does not include a documented systematic review of the literature with meta-analysis of RCTs**

If the publication does not include a documented systematic review of the literature, does not quantitatively synthesize the results of at least 2 RCTs, or includes non-RCTs in a meta-analysis, then it is excluded.

**No: RCTs included in the meta-analysis are not of pharmacological intervention with single drug or class of drug compared against an alternative treatment.**

If the meta-analysis does not include RCTs that evaluate the efficacy or harm of a drug or class of drug against an alternative treatment (e.g., placebo, alternative drug) then it is excluded. Meta-analyses that only compare administration or dosing methods of the same drug are excluded. Meta-analyses of interventions where a drug may be administered but is not central to the intervention are not included (e.g., collaborative depression care). Publications with meta-analyses that investigated a combination of pharmacological and non-pharmacological interventions (e.g., psychotherapy) or interventions which may or may not involve a drug (e.g., amnioinfusion) are only included if a study group was exclusively given a drug intervention or if the meta-analysis assessed the addition of a drug to a treatment received by both intervention and control groups. Drugs are defined broadly to include biologics and vaccines, but not nutritional supplements (e.g., vitamins) or medical devices without a drug component. A product is considered to be a drug if it was assigned either a New Drug Application (NDA) or Abbreviated New Drug Application Number (ANDA) FDA application number, based on the Drugs@FDA database, indicating a brand name or generic drug application, respectively. If a product cannot be found in the Drugs@FDA database, its status as a drug will be determined by expert consensus. Reviews which only compare different dosages of the same drug or different methods of administration of the same drug are excluded.

**No: Not RCTs of pharmacological interventions that include at least one drug under patent at the time of publication.**

If the review does not include at least one drug in the intervention or comparison study arms that was under patent in the United States (U.S.) at the time of publication based on the electronic U.S. Food and Drug Administration (FDA) Orange Book. A drug was considered to be under patent if any aspect of the active ingredient (e.g., dosage, route, strength) was protected by an unexpired patent.

Full Text:

**Does this study meet the coding criteria for inclusion?**

**No: Does not include a documented systematic review of the literature with meta-analysis of RCTs**

If the publication does not include a documented systematic review of the literature, does not quantitatively synthesize the results of at least 2 RCTs, or includes non-RCTs in a meta-analysis, then it is excluded.

**No: RCTs included in the meta-analysis are not of pharmacological intervention with single drug or class of drug compared against an alternative treatment.**

If the meta-analysis does not include RCTs that evaluate the efficacy or harm of a drug or class of drug against an alternative treatment (e.g., placebo, alternative drug, other non-drug comparator) then it is excluded. Meta-analyses that only compare administration or dosing methods of the same drug are excluded. Meta-analyses of interventions where a drug may be administered but is not central to the intervention are not included (e.g., collaborative depression care). Publications with meta-analyses that investigated a combination of pharmacological and non-pharmacological interventions (e.g., psychotherapy) or interventions which may or may not involve a drug (e.g., amnioinfusion) are only included if a study group was exclusively given a drug intervention or if the meta-analysis assessed the addition of a drug to a treatment received by both intervention and control groups. Drugs are defined broadly to include biologics and vaccines, but not nutritional supplements (e.g., vitamins) or medical devices without a drug component. A product is considered to be a drug if it was assigned either a New Drug Application (NDA) or Abbreviated New Drug Application Number (ANDA) FDA application number, based on the Drugs@FDA database, indicating a brand name or generic drug application, respectively. If a product cannot be found in the Drugs@FDA database, its status as a drug will be determined by expert consensus. Reviews which only compare different dosages of the same drug or different methods of administration of the same drug are excluded.

**No: Not RCTs of pharmacological interventions that include at least one drug under patent at the time of publication.**

If the review does not include at least one drug in the intervention or comparison study arms that was under patent in the United States (U.S.) at the time of publication based on the electronic U.S. Food and Drug Administration (FDA) Orange Book. A drug was considered to be under patent if any aspect of the active ingredient (e.g., dosage, route, strength) was protected by an unexpired patent.

**Yes:** Study eligible to be included.
