# Supplementary material for: Reporting of financial conflicts of interest in meta-analyses of drug trials published in high-impact medical journals: comparison of results from 2017 to 2018 and 2009
Source: Syst Rev. 2020 Apr 8;9:77. doi: 10.1186/s13643-020-01318-5 (PMC7140556; doi:10.1186/s13643-020-01318-5)
Supplement: Supplementary file 2 — Additional file 2:. Methods 2. Meta-Analysis Data Extraction. [file 13643_2020_1318_MOESM2_ESM.docx]

**Additional Methods 2. Meta-Analysis Data Extraction**

- First Author, last name
- Year of publication (or in press)
- Journal
- Cochrane Review (Y/N)
- **# of RCTs synthesized in Meta-Analysis**
- Date Range of Included Studies
- Study population
- Pharmacological agent
- Control/comparison arms
- Outcome (efficacy, harm, or both efficacy & harm)
- Meta-Analysis Author Financial Ties / Funding Sources Reported
- Funding Source of Meta-Analysis
- Type of Industry Funding # of Meta-Analysis Authors
- # Meta-Analysis Authors with financial ties
- # Meta-Analysis Authors with Current Industry Affiliation (Employment)
- Type of Meta-analysis Author Financial Ties # Meta-Analysis Authors with Former Industry Affiliation (Employment)
- Type of Meta-analysis Author Financial Ties: # Authors industry board members
- Type of Meta-analysis Author Financial Ties: # Authors industry consultants
- Type of Meta-analysis Author Financial Ties: # Authors report equity
- Type of Meta-analysis Author Financial Ties: # Authors provide expert testimony
- Type of Meta-analysis Author Financial Ties: # Authors receive gifts
- Type of Meta-analysis Author Financial Ties: # Authors hold patents
- Type of Meta-analysis Author Financial Ties: # Authors receive payment for manuscript preparation
- Type of Meta-analysis Author Financial Ties: # Authors receive research funding from industry
- Type of Meta-analysis Author Financial Ties: # Authors receive royalties
- Type of Meta-analysis Author Financial Ties: # Authors receive speaker fees/payment for the development of presentations from industry
- Type of Meta-analysis Author Financial Ties: # Authors receive travel reimbursement from industry
- Type of Meta-analysis Author Financial Ties: # Authors have unspecified FCOIs from industry
- Meta-analysis Authors Report Funding Sources of Included Studies (Y/N)
- Reporting Style of Included Study Funding Sources
- Meta-analysis Authors Report Author Financial Ties of Included Studies (Y/N)
- Reporting Style of Included Study Author Financial Ties
- Meta-analysis Authors Report Author-industry Affiliation (Employment) of Included Studies (Y/N)
- Reporting Style of Included Study Author-industry Affiliation
- Quality or Risk Assessment of Included RCTs (Y/N)
- Quality or Risk Assessment Method of Included RCTs
- Inclusion and Placement in publication of Included RCTs’ Funding Sources Reported in Quality or Risk Assessment Method (Y/N)
- Inclusion and Placement in Publication of Included RCTs’ Author Financial Ties (including former and current industry employment)
- Reported in Quality or Risk Assessment Method (Y/N)
- Placement in Publication of Included RCTs’ Funding Sources Reported
- Placement in Publication of Included RCTs’ Author Financial Ties Reported
